# Multiple lineage specific expansions within the guanylyl cyclase gene family

**DOI:** 10.1186/1471-2148-6-26

**Published:** 2006-03-20

**Authors:** David A Fitzpatrick, Damien M O'Halloran, Ann M Burnell

**Affiliations:** 1 Biology Department, National University of Ireland Maynooth, Maynooth, Co. Kildare, Ireland; 2 School of Biomolecular and Biomedical Science, Conway Institute, University College Dublin, Belfield, Dublin 4, Ireland; 3 Center for Neuroscience, UC Davis, 1544 Newton Ct., Davis, CA 95616, USA

## Abstract

**Background:**

Guanylyl cyclases (GCs) are responsible for the production of the secondary messenger cyclic guanosine monophosphate, which plays important roles in a variety of physiological responses such as vision, olfaction, muscle contraction, homeostatic regulation, cardiovascular and nervous function. There are two types of GCs in animals, soluble (sGCs) which are found ubiquitously in cell cytoplasm, and receptor (rGC) forms which span cell membranes. The complete genomes of several vertebrate and invertebrate species are now available. These data provide a platform to investigate the evolution of GCs across a diverse range of animal phyla.

**Results:**

In this analysis we located GC genes from a broad spectrum of vertebrate and invertebrate animals and reconstructed molecular phylogenies for both sGC and rGC proteins. The most notable features of the resulting phylogenies are the number of lineage specific rGC and sGC expansions that have occurred during metazoan evolution. Among these expansions is a large nematode specific rGC clade comprising 21 genes in *C. elegans *alone; a vertebrate specific expansion in the natriuretic receptors GC-A and GC-B; a vertebrate specific expansion in the guanylyl GC-C receptors, an echinoderm specific expansion in the sperm rGC genes and a nematode specific sGC clade. Our phylogenetic reconstruction also shows the existence of a basal group of nitric oxide (NO) insensitive insect and nematode sGCs which are regulated by O_2_. This suggests that the primordial eukaryotes probably utilized sGC as an O_2 _sensor, with the ligand specificity of sGC later switching to NO which provides a very effective local cell-to-cell signalling system. Phylogenetic analysis of the sGC and bacterial heme nitric oxide/oxygen binding protein domain supports the hypothesis that this domain originated from a cyanobacterial source.

**Conclusion:**

The most salient feature of our phylogenies is the number of lineage specific expansions, which have occurred within the GC gene family during metazoan evolution. Our phylogenetic analyses reveal that the rGC and sGC multi-domain proteins evolved early in eumetazoan evolution. Subsequent gene duplications, tissue specific expression patterns and lineage specific expansions resulted in the evolution of new networks of interaction and new biological functions associated with the maintenance of organismal complexity and homeostasis.

## Background

Guanylyl cyclases (GCs) are responsible for the production of the secondary messenger cyclic guanosine monophosphate (cGMP). cGMP plays important roles in a variety of physiological responses such as vision, olfaction, muscle contraction, homeostatic regulation, cardiovascular and nervous function [1]. GCs are multi-domain proteins, which occur in two forms: receptor guanylyl cyclases (rGCs) and soluble guanylyl cyclases (sGCs). The phylogenetic relationships among GC isoforms of vertebrates and invertebrates have not been thoroughly investigated. We have used whole genome sequence data to investigate the evolution of GCs across a diverse range of animal phyla. Our analyses reveal that the GC family has undergone several lineage specific gene expansions, most notably in nematodes, echinoderms and vertebrates.

rGCs were first isolated in the echinoderms, where they are involved in chemotaxis between egg and sperm cells [2]. Seven different classes of rGC genes have been found in mammals, each represented in humans by a single gene. Two of these genes (GC-D and GC-G), are considered to be pseudogenes in humans [3], while the remaining five genes encode functional rGCs. Two of these, GC-A and GC-B, are targets for the natriuretic peptides – a family of polypeptide hormones that act to reduce blood volume by stimulating natriuresis and diuresis in the kidney [4]. The GC-C receptor was first described as the target for heat-stable enterotoxin secreted by pathogenic strains of *Escherichia coli *[5]. GC-C is expressed in the intestine where it is involved in the regulation of fluid and electrolyte balance and its endogenous ligands have been identified as uroguanylin and guanylin [6]. Retinal rGCs (GC-E and GC-F) play a critical role in vision, as they enhance the synthesis of cGMP in a negative Ca^2+ ^modulated feedback loop. These retinal rGCs are regulated by small Ca^2+^-binding proteins that detect changes in cytoplasmic Ca^2+ ^concentration and act through the cytoplasmic domain of the protein [7]. Homologues of all the mammalian rGCs have also been detected in teleost fish [8].

The genome of *Drosophila melanogaster *contains six predicted rGC genes and the *Anopheles *genome is predicted to have an orthologue of each of these six genes [9]. One rGC gene has been cloned from the tobacco hornworm *Manduca sexta *[10] and one from the silkmoth *Bombyx mori *[11]. The physiological roles of rGCs in insects are not well characterised, but it has been established that the *B. mori *rGC, BMGC-1 is regulated in the flight muscles in a circadian fashion [12]; that the *M. sexta *neural-specific rGC, MSGC-II is most similar to the vertebrate retinal guanylyl cyclases and is inhibited by Ca^2+ ^[13] and that the rGC protein, Gcy76C, is required for axonal repulsion in *D. melanogaster *[14]. Thus it seems likely that in insects, as in vertebrates, rGCs play important roles in a variety of physiological responses. The *Caenorhabditis elegans *genome contains at least 25 predicted rGC genes [15,16]. The expression patterns often of these genes have been investigated, and all ten are expressed in subsets of *C. elegans *sensory neurons. Mutant phenotypes have been described for two of these rGC genes: *odr-1 *[16] and *daf-11 *[17] and in both cases chemosensory signalling is affected.

**Figure 1:**
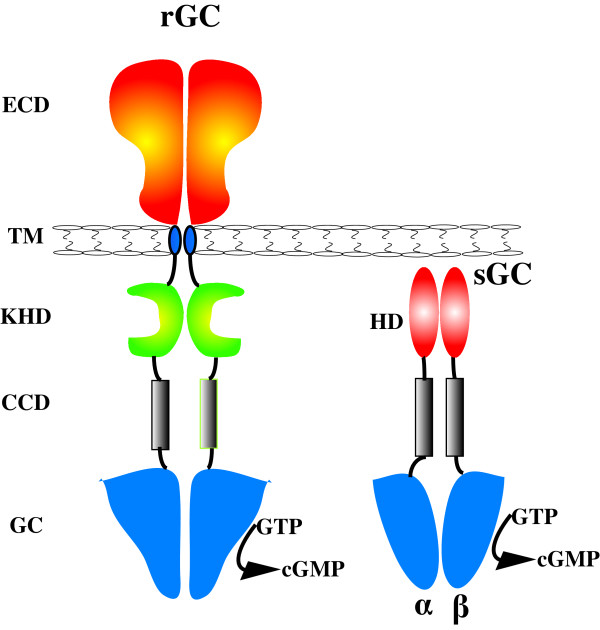
**General domain structure of dimeric receptor and soluble guanylyl cyclases. **The receptor architecture consists of the extracellular domain(BCD), transmembrane segment (TM), kinase homology domain (KHD), coiled-coil domain (CCD) and guanylyl cyclase domain (GC). The heterodimeric soluble guanylyl cyclase consists of a heme domain, a CC and a GC domain.

rGCs contain an extracellular binding domain, a single membrane-spanning domain, a protein kinase homology domain (KHD) and intracellular coiled-coil dimerization and catalytic domains (Figure 1). The KHD has significant similarity with known protein kinases but contains no kinase activity; it functions as a negative regulatory element whose deletion by mutagenesis gives rise to a constitutively active GC receptor [18]. The region between the cyclase domain and the KHD forms a coiled-coil domain, enabling the formation of dimeric proteins which are considered to be the minimal catalytic unit for human rGC enzymes [19].

sGCs are ubiquitously expressed in mammalian cells where they affect a variety of important physiological functions including smooth muscle relaxation, vasodilation, neuronal signal transduction, blood platelet reactivity and phototransduction [20]. They are activated by nanomolar concentrations of nitric oxide (NO), a freely diffusible membrane permeant gas. In mammals sGC typically forms a heterodimer composed of an α- and a β-subunit, each of which contains a regulatory domain, a coiled-coil domain and a cyclase domain (Figure 1). The human genome encodes two sGC α-subunit and two sGC β-subunit genes. The N-terminal portion of the β-subunit constitutes the heme-binding domain that confers NO sensitivity to the enzyme. Upon activation of the sGC by NO the GC activity is accelerated by 100–300 fold [21]. At the C-terminus of each subunit is a well-conserved catalytic domain. Contained between the heme-binding and catalytic regions is a dimerization domain responsible for heterodimer formation. A prokaryote heme binding protein family with significant sequence identity to the heme binding domain of eukaryotic sGCs has been identified [22]. This heme-binding family was found in various bacterial lineages, but among the eukaryotes was detectable only in the animal lineage. Among the residues conserved in the prokaryote sequences are the histidine residue which covalently binds the heme prosthetic group and a YxS|TxR motif which has also been implicated in heme binding [23]. sGC heme-like domains from the obligate anaerobe *Thermoanaerobacter tengcongenesis *and the facultative anaerobe *Vibrio cholerae *have been cloned [24]. That study found that *V. cholerae *protein bound NO, whereas the *T. tengcongenesis *protein is capable of forming a stable O_2 _complex and has NO binding characteristics similar to myoglobin and other O_2 _sensors This heme-binding family has therefore been named H-NOX (Heme-Nitric oxide/Oxygen binding) [24] and is made up of two domains, the H-NOB (Heme NO Binding) and H-NOBA (Heme NO Binding Associated) [22]. The H-NOBA domain occurs between the HNOB and the cyclase domains in animal sGCs [22].

The aim of the work reported here was to utilise whole genome data from vertebrate and invertebrate animals to describe a molecular phylogeny for both the soluble and receptor GCs. The most notable features of the resulting phylogenies are the number of lineage specific rGC and sGC expansions that have occurred during metazoan evolution. These lineage specific expansions have resulted in great diversity within the signal transduction and cellular communication pathways which are regulated by this functionally diverse multi-domain GC protein family.

## Results

### Receptor GC phylogeny

**Figure 2:**
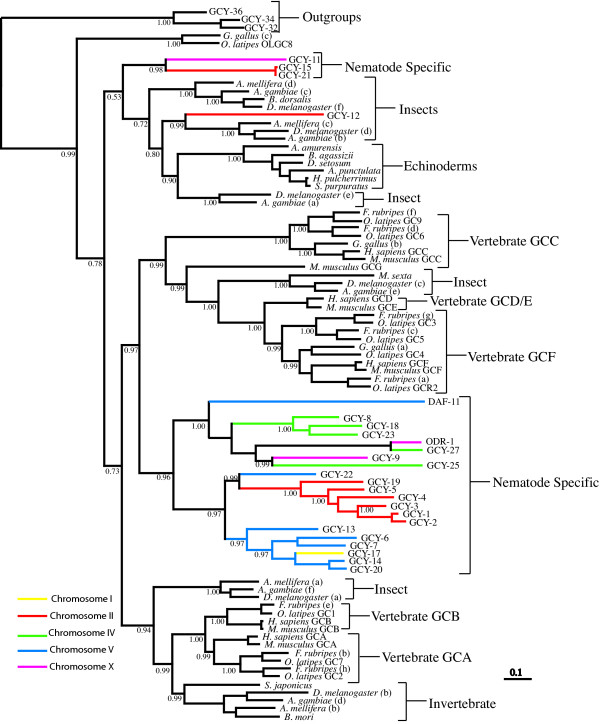
**Phylogenetic analysis of receptor guanylyl cyclases. **Inferred phylogeny of the receptor guanylyl cyclase gene family using a Bayesian consensus tree derived from amino acid alignments constructed using MRBAYES 3.0B4 [80]. The soluble guanylyl cyclase genes; *gcy-36*, *gcy-34 *and *gcy-32 *from *C. elegans *are used as an outgroup. *C. elegans *guanylyl cyclase gene branches are color coded to correspond with chromosomal linkage.

The most striking aspect of our phylogenetic reconstruction for the rGC gene family represented in Figure 2, is that the majority (21 out of 25) of the *C. elegans *rGC genes form a robust lineage-specific clade with strong support (0.96 Bayesian posterior probability (BPP)). According to our phylogenetic hypothesis this nematode group is a sister clade to a large strongly supported (0.97 BPP) clade of rGC genes containing vertebrate GC classes (retinal rGC receptors, sensory organ rGCs and the enterotoxin/guanylin rGCs) and a group of insect rGCs. The vertebrate atrial natriuretic peptide receptors form a distinct clade (1.00 BPP). Sequence similarity between the GC-A and GC-B natriuretic receptors is high and these rGCs group together as sister taxa. The natriuretic receptors may share a common ancestor with two insect clades as these sequences are grouped together with relatively high support (0.94 BPP). Also included as a sister group to one of the insect clades is a single rGC sequence from the echinoderm *Stichopus japonicus*. These observations suggest that natriuretic rGC regulation may have originated from basal invertebrates after nematode divergence, as no *C. elegans *orthologues are found within this clade. An alternative hypothesis is that *C. elegans *may have lost these natriuretic receptors but this would appear to be a less parsimonious explanation.

Of the remaining nematode rGC sequences, GCY-11, GCY-15 and GCY-21 group together with strong support (0.98 BPP). This small nematode clade is located beside insect and echinoderm sperm rGCs. The supports for these inferences are weak (0.52 BPP), but it is evident that this nematode rGC group is highly divergent and it may be evolving at a faster rate when compared to the other nematode rGC sequences and indeed with the other rGC genes in this dataset. The remaining nematode rGC sequence, GCY-12, is grouped beside an insect clade with strong support (0.99 BPP) and this insect/nematode clade is positioned beside the echinoderm sperm-specific clade, but with relatively weak support (0.80 BPP). The echinoderm sperm-activating rGC sequences included in this analysis group together in a single lineage-specific clade with maximum support (1.00 BPP).

**Figure 3:**
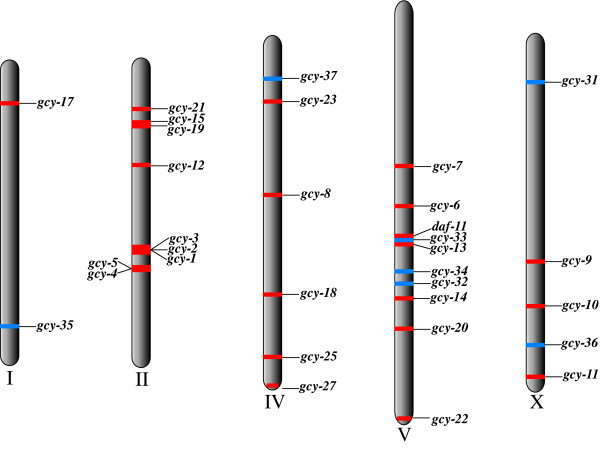
**Genomic localisation of all *C. elegans *guanylyl cyclase genes. **Soluble GCs are denoted in blue while receptor GCs are red. Receptor GCs are represented on all chromosomes, although there appears to be intrachromosomal duplication on chromosomes II, IV and V. Soluble GCs are represented on chromosomes I, IV, V and X.

We investigated the chromosomal locations of the *C. elegans *rGC genes and compared these with their phylogenetic positions. We observed large clusters of genes from the major *C. elegans *specific clade on chromosomes II, IV and V (Figure 3). Our analysis revealed that there is a general correlation between the chromosomal and phylogenetic positions of these genes, implying that the *C. elegans *rGC gene expansion resulted from intrachromosomal gene duplications. There are some exceptions, for example *gcy-9 *and *odr-1 *are located on chromosome X but they are grouped phylogenetically with *gcy *genes located on chromosome IV; similarly *gcy-17 *is located on chromosome I and is most closely related to genes located on chromosome V, and *gcy-22*, which is located on chromosome V groups with genes from chromosome II. The incidences where phylogenetic and chromosomal positions are not congruent are most probably the result of interchromosomal recombination.

### EST database searches

To investigate the possibility of finding orthologues of *C. elegans *lineage specific GC proteins throughout the Phylum Nematoda we performed a database search of the nematode EST database NEMBASE and the *Brugia malayi *genome from The Institute of Genomic Research (TIGR). From this analysis we identified 30 matches from 15 different nematode species, which span four of the five major clades of the Phylum Nematoda for which sequence data is available (Table 1). These 30 matches contain orthologues of the *C. elegans *receptor and soluble GC genes. This finding implies that the distinct groups of *C. elegans *GC genes identified in our phylogenies are not specific to *C. elegans *alone but are common to all nematodes, a finding that will only be verified when more whole nematode genomes become available. Orthologues of all *C. elegans *GC genes were located within the *C. briggsae *genome. No homologues of the nematode specific GC genes were detected in the Schistosome or Tardigrade EST databases.

**Table 1:** Nematode EST table. Blast matches of *C. elegans *guanylyl cyclase in the four major clades of the phylum Nematoda in Nembase. Accesion number correspond to those in NEMBASE.

Clade	**Nematode Species**	**Nematode Specific Guanylyl Cyclase Genes**	Accession Numbers
I	Trichuris vulpis	gcy-14	TVC00239

III	*Ascaris suum*	*gcy-27*	ASC22837
			
	*Brugia malayi*	*gcy-12*	14378.m00176
		*gcy-18*	14977.m05059
		*gcy-35*	14232.m00262
		*odr-1*	14958.m00342

IVa	*Strongyloides ratti*	*gcy-6*	SRC05888
		*gcy-23*	SRC01902
			
	*Parastrongiyoides trichosuri*	*gcy-6*	PTC00967

IVb	*Globodera rostochiensis*	*gcy-9*	GRC01455
		*gcy-18*	GRC01798
		*gcy-22*	GRC03191
			
	*Heterodera glycines*	*gcy-9*	HGC00473
		*gcy-20*	HGC10067
		*gcy-27*	HGC02658
			
	*Meloidogyne arenaria*		
		*gcy-12*	MAC01982
		*gcy-13*	MAC03089
			
	*Meloidogyne incognita*		
		*gcy-7*	MIC03007
		*gcy-9*	MIC02891
	*Meloidogyne javanica*		
		*gcy-1*	MJC01228
		*gcy-23*	MJC04228
	*Meloidogyne chitwoodi*		
		*gcy-6*	MCC03766
		*gcy-13*	MCC02926
	*Meloidogyne hapla*		
		*gcy-9*	MHC10317
		*gcy-18*	MHC00708
	*Heterodera schachtii*		
		*gcy-22*	HSC01152
		*gcy-23*	HSC00122
	*Ancylostoma caninum*	*gcy-37*	ACC02290

V	*Anycylostoma ceylanicum*	*gcy-37*	AYC04102
		*gcy-8*	AYC03974

### Partial cyclase domain phylogeny with particular reference to echinoderms

**Figure 4:**
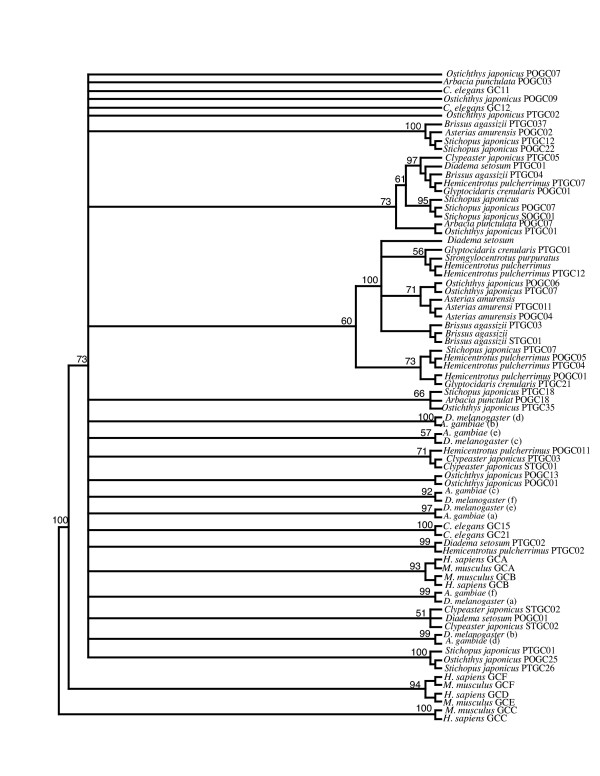
**Phylogenetic tree derived from partial cyclase domain. **Bootstrap supports are shown for select nodes. Large number of polytomies are the result of the cyclase domain being relatively short and highly conserved. Echinoderm sequence data were taken from [73] and we have maintained their notation. A select number of sequences present in Figure [2] are also included in this phylogeny.

A previous study has shown that echinoderms have many diverse guanylyl cyclase isoforms [25]. However the sequence data used in that study contained only 121 amino acid positions from the highly conserved cyclase domain. To investigate the relationships between these echinoderm sequences and our dataset, we aligned the corresponding region of the rGC cyclase domain of all human, mouse, *D. melangoster *and *A. gambiae *and selected nematode genes with the echinoderm sequences. Examination of the phylogenetic tree derived from this alignment showed a large number of polytomies (Figure 4). This result is unsurprising, as the alignment is quite short and highly conserved. Consequently the alignment lacks enough phylogenetic information to infer deep branching relationships. There are a small number of highly supported clades however. For example individual vertebrate classes are grouped beside one another with relatively high support (93% and 94% bootstrap support respectively). Similarity particular isoforms from the same echinoderm species are found grouped together. For example *Asterias amurensis*, *Brissus agassizii*, *Stichopus japonicus *and *Hemicentrotus pulcherrimus *isoforms are grouped together (Figure 4). There does not appear to be any differentiation between rGC sequences isolated from the testes or ovaries of these echinoderm species, thus there is no evidence for organ specific clades. For example *A. amurensis *rGC sequences isolated from the testes and ovaries are grouped within the same clade to the exclusion of other rGC sequences. Based on our phylogeny we can say that there are lineage specific expansions of guanylyl cyclases within the echinoderms, however we do not have enough sequence data at our disposal to properly quantify these expansions.

### Soluble GC phylogeny

**Figure 5:**
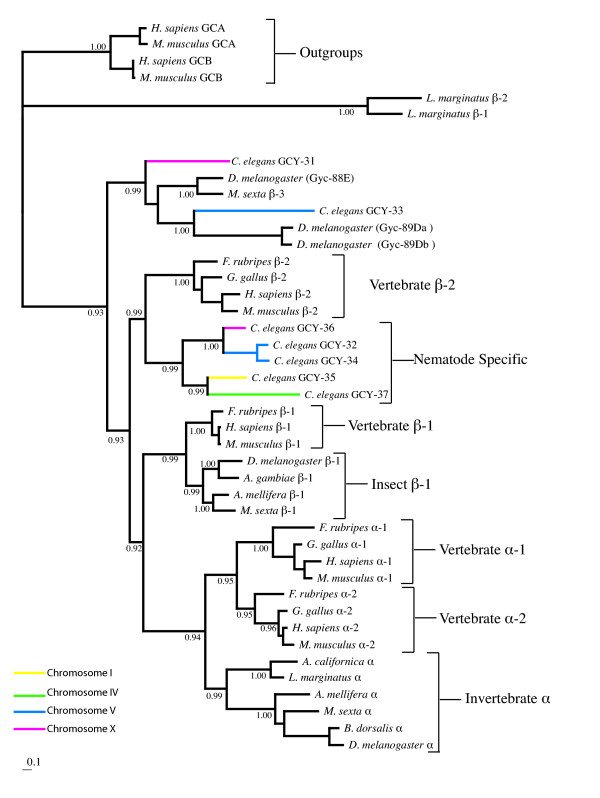
**Phylogenetic analysis of soluble guanylyl cyclases. **Inferred phylogeny of the soluble guanylyl cyclase gene family using a Bayesian consensus tree derived from amino acid alignments constructed using MRBAYES 3.0B4 [80]. Posterior probabilities for selected branches are shown at nodes. The receptor guanylyl cyclase orthologues GC-A and GC-B from human and mouse are used as outgroups. The scale bar indicates number of changes per site. *C. elegans *guanylyl cyclase gene branches are color coded to correspond with chromosomal linkage.

The sGC genes of vertebrates and nematodes have undergone a less extensive gene expansion than the rGC genes, resulting in a smaller sGC dataset. Interestingly the sGC phylogeny also reveals a highly supported (0.99 BPP) *Caenorhabditis *specific clade (Figure 5), which contains five of the seven *C. elegans *sGCs. The *C. elegans *sGC genes are located on chromosomes I, IV, V and X (Figure 5). With the exception of *gcy-32 *and *gcy-34 *which both reside on chromosome V and group together on our phylogenetic tree, no other relationship between phylogenetic position and chromosomal linkage was observed for the *C. elegans *sGC family. The *C. elegans *specific sGC group is a sister clade (0.99 BPP) to the vertebrate β-2 sGCs. The two vertebrate α classes (α-1 and α-2) form a robust clade (0.95 BPP) and the insect α genes form a highly supported (0.99 BPP) sister clade to these vertebrate α-sGC classes. Grouped with the insect α-sGC clade are sequences from two mollusc taxa (*Limax marginatus *and *Aplysia californica*). All of these vertebrate, insect and mollusc α class sGCs are contained within a large robust clade (0.94 BPP). The insect β-1 genes form a distinct group which is a sister clade to the vertebrate β-1 genes (0.99 BPP). According to our inference the vertebrate β-1 class is more closely related to the vertebrate α classes than to the vertebrate β-2 class, as it forms a sister clade (0.92 BPP) to the group containing the vertebrate and invertebrate α sGC sequences.

Two sGC sequences from *C. elegans*, GCY-31 and GCY-33, group with atypical insect sGCs which have a reduced affinity for NO. One of these sGCs is the *M. sexta *β-3 protein, which has been shown to lack two cysteine residues important for NO sensitivity [13,26]. The other three *D. melanogaster *sGC proteins (Gyc-88E, Gyc-89Db and Gyc-89Db) in this clade also display weak NO binding capabilities and function as molecular oxygen sensors [27]. *C. elegans *sGC genes also lack critical aa residues required for NO activation in mammalian sGC β-subunits [28] and the sGC, GCY-35 from *C. elegans *binds molecular oxygen [29]. The grouping of these nematode sGCs with an NO insensitive insect GC clade at the base of the sGC phylogenetic tree suggests that NO regulation by sGCs may be an evolutionary novelty, which occurred early in metazoan evolution. Since the insects *M. sexta *and *D. melanogaster *also contain NO sensitive sGC subunits (located as a sister clade to the vertebrate β-1 genes), the origin of NO regulation by sGCs most probably postdates the divergence of nematodes but predates arthropod divergence.

### H-NOX phylogeny and alignment

**Figure 6:**
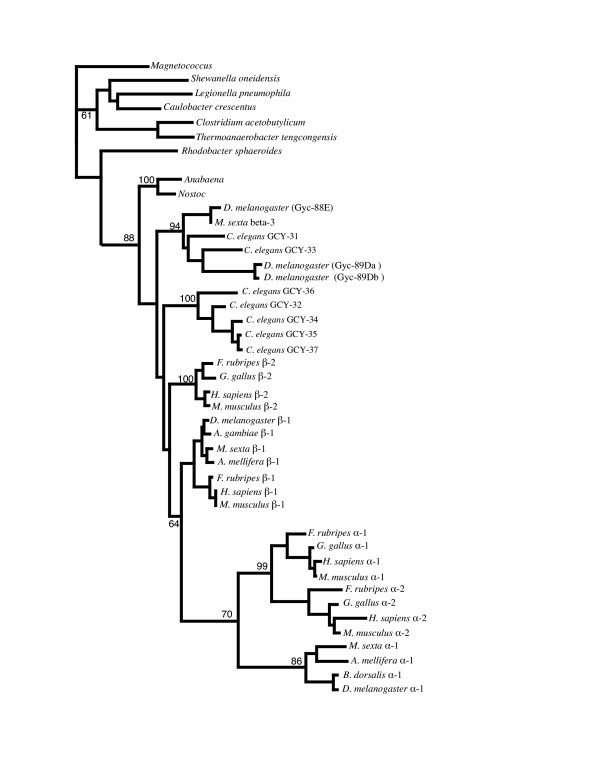
**Phylogenetic analysis of heme nitric oxide binding (H-NOB) domain.**This phylogeny was created using the H-NOB domain from the soluble guanylyl cyclases in Figure 5 and a number of bacterial sequences containing the H-NOB domain. Bootstrap supports are shown for select nodes. In agreement with [22] the cyanobacteria (*Nostoc *and *Anabaend*) are inferred to be ancestral to all animal guanylyl cyclases (88% bootstrap support).

**Figure 7:**
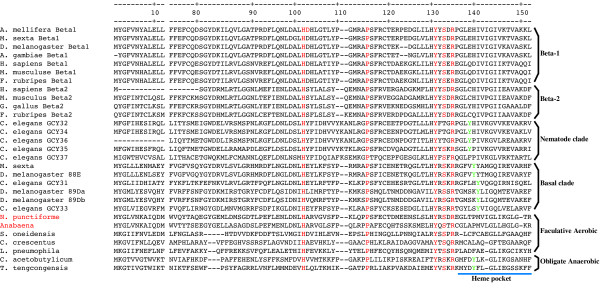
**Partial multiple sequence alignment of selected eukaryotic and prokaryotic H-NOB proteins. **Numbering on top corresponds to the H-NOB domain of *T. tengcongensis*. Residues conserved among all H-NOB proteins and important for heme binding are highlighted in red. The Tyr-140 residue, which is essential for the stabilisation of O_2 _binding in *T. tengcongensis *is highlighted is green. The names of the two cyanobacterial sequences present are hightlighted in red.

Phylogenetic analysis suggests that the H-NOB domains from *Nostoc *and *Anabaena *were the most closely related prokaryotic sequences to the animal sGC heme binding domain [22]. Our phylogenetic tree of H-NOB domain sequences from animal sGCs and various bacterial lineages (Figure 6) confirms this observation and also supports the postulate that the sGC H-NOB domain was acquired by horizontal transfer from a cyanobacterial source [22]. Sequence analysis of the H-NOX family from anaerobic bacteria (which are predicted to bind O_2_, based on results with *T. tengcongensis*) identified three conserved residues, Trp-9, Asn-74 and Tyr-140, which were absent from the H-NOB domains of facultative aerobes and vertebrate sGCs that do not bind O_2 _[30]. Using mutational analysis it was found that Tyr-140 was essential for the stabilisation of O_2 _binding in *T. tengcongensis *H-NOB [31]. The crystal structure of *T. tengcongensis *H-NOB shows that Tyr-140 is located within the distal part of the heme pocket and is the only polar residue in the lining of the distal heme pocket [32]. It has also been shown that the introduction of a Tyr residue into the non-polar distal heme pocket of the following H-NOB domains: *Legionella pneumophila *(at position 142) and rat β-1 sGC (at position 145) results in proteins with acquired capacity for binding O_2 _[31]. A multiple sequence alignment of the H-NOB domains from the basal sGC clade and the nematode specific sGC clade with prokaryote H-NOB sequences is shown in Figure 7. This alignment shows the high degree of sequence conservation between cyanobacterial H-NOB sequences and the sequences in the basal animal sGC clade. All H-NOB sequences share the following conserved residues: His-102 which is the proximal ligand for the heme iron [32,33]; Pro-115 which makes hydrophobic contact with the heme pyrolle D ring [32]; Ile [Val|Leu]-5 which makes hydrophobic contact with the pyrolle A ring [32] and the YxS|TxR motif (Tyr-131, Ser-133, Arg-135) which is involved in hydrogen bonding interactions with the propionate groups of the heme protophorphyrin [23,32]. Interestingly our alignment also shows that in the predicted distal pocket region sequences from the O_2 _sensitive basal sGC clade have a Tyr residue at position 141 while the nematode specific clade has Tyr at position 138 (with the exception of GCY 37 which has Phe-138). However all β-1 and β-2 sGC sequences, which bind NO, lack a Tyr residue in the predicted distal pocket region, and have a non-polar I1e residue at position 141. Substitution of I1e-141 by Tyr-141 in the rat β-1 sGC changed its ligand binding specificity from NO to O_2 _[30].

## Discussion

Guanylyl- and adenylyl cyclases are multi-domain proteins that display a wide range of structural forms. Prokaryotes possess six classes (I-VI) of adenylyl cyclases (ACs), five of which exhibit a variety of structurally unique catalytic domains that are absent from eukaryotes [34]. The class III purine nucleotide cyclase domain is present in all eukaryotes, however the diversity observed in eukaryotes for this class is only a small subset of the class III multi-domain cyclase proteins found in prokaryotes [35]. In prokaryotes GCs are less abundant than ACs. To date only a single candidate prokaryote GC gene has been found (*Cya2*) in the cyanobacterium *Synechocystis *[36]. Some authors postulate that the relative stability of cGMP, in comparison to cAMP, may have contributed to the absence of cGMP in bacteria, where a rapid turn-over of signalling molecules is required [35]. There is no evidence for GC genes in any of the complete yeast genomes, nor have they been reported so far in other fungi [34]. Similarly, while class III cyclases appear to be abundant in the chlorophyte algal antecedents of land plants, they are absent in flowering plants [34]. Five class III purine nucleotide cyclase genes have been detected in the *Dictyostelium discoideum *[37]. Class III cyclases have also been detected in several other phylogenetically divergent protistan phyla, however the diversity of structural forms displayed by these cyclases suggests that they are of paraphyletic origin [34,38]. The catalytic domains of class III ACs and GCs from metazoans appear to be monophyletic [38,39]. Functional similarities between the catalytic domains of metazoan ACs and GCs have also been demonstrated. For example, targeted replacement of two amino acids in the guanine-binding pocket of the rat retinal GC-1 receptor changed its specificity from GTP to ATP, while retaining its capacity to be activated by Ca^2+^-binding proteins [40].

Based on whole genome data and individual studies it is apparent that rGCs are a highly successful and diverse protein family which are utilised by vertebrate and invertebrate animals for a variety of roles in signal transduction and organismal homeostasis. The accretion of additional domains to the class III cyclase domain (especially the extracellular receptor, transmembrane and regulatory domains, Figure 1) has resulted in a very successful multi-domain rGC protein. This modular arrangement has facilitated the evolution of novel physiological rGC signalling pathways in different animal lineages through modifications to the receptor and regulatory domains. Subsequent gene duplications have further expanded and refined these pathways leading to several lineage specific gene expansions of rGC genes. Among these expansions is a large nematode specific rGC clade comprising 21 genes in *C. elegans *alone; a vertebrate specific expansion in the natriuretic receptors GC-A and GC-B; a distinct vertebrate specific expansion in the guanylyl GC-C receptor and an echinoderm specific expansion in the sperm rGC genes. Similar domain accretions and lineage expansions have also occurred in the sGCs, but the intracellular localisation of sGCs restricts the diversity of extracellular ligands which can activate them to membrane permeant molecules such as NO and O_2_. Despite this, NO is an important signalling molecule involved in the regulation of diverse physiological mechanisms in the vertebrate cardiovascular, nervous and immune systems. The synthesis of NO by nitric oxide synthases (NOS) is controlled by complex intrinsic and extrinsic factors such as post translational modification, co-factor and substrate compartmentalization, phosphorylation and specific interactions with other proteins such as calmodulins [41,42].

The *D. melanogaster *genome contains five genes encoding sGC subunits. Two of these genes, Gycα-99B and Gycβ-100B, encode α- and β-subunits which form a conventional heterodimeric NO-sensitive sGC, whereas the remaining three genes encode subunits with reduced sensitivity to NO [43]. An sGC subunit from *Manduca sexta *was the first example of a sGC which exhibited enzyme activity without the need for co-expression of additional subunits. This *M. sexta *sGC is insensitive to NO [26] and forms active homodimers [44]. The genome of *C. elegans *contains seven predicted sGC genes, but unlike *D. melanogaster*, a nitric oxide synthase gene has not been detected in the *C. elegans *genome [45]. The sequences of all seven *C. elegans *sGCs are more similar to the β-subunits of mammalian guanylyl cyclases than to the α-subunits. However, while the *C. elegans *sequences conserve the heme-binding histidine residue, they lack two critical cysteine residues required for NO activation in mammalian sGC β-subunits [28]. GCY-35 is required by *C. elegans *for avoiding hyperoxic conditions and unlike canonical NO-sensitive sGCs, it can bind oxygen [29]. Similarly the *D. melanogaster *atypical sGCs have also been shown to function as molecular oxygen sensors [27].

In invertebrates with external fertilization, chemotaxis is a key event in guiding sperm to conspecific eggs. The jelly coat of echinoderm eggs releases species specific sperm-activating peptides [46] and echinoderm sperm have specific rGCs for these chemotactic molecules. Activation of sperm rGCs leads to a rapid, large but transient rise in cGMP and mediates ion fluxes across the sperm membrane. This in turn affects flagellar motion and the direction of movement [47,48]. According to our phylogenetic hypothesis (Figure 2) echinoderm sperm rGCs form a distinct lineage-specific clade within a larger clade of invertebrate rGCs. Whether other invertebrates with external fertilization use rGC signalling has not been established. In mammals with internal fertilization sperm chemotaxis is also a critical component of the fertilisation process, but here chemotactic responses depend on G protein coupled chemoreceptors. Interestingly, evidence is accumulating that sperm maturation and the acrosome reaction are induced in mammalian sperm by stimulation of an NO-sensitive sGC [49].

The natriuretic receptors GC-A and GC-B appear to be a vertebrate specific novelty – each represented by a single gene in mammals. Their ligands, the natriuretic peptides (NP), also comprise a small protein family. Available data indicate that there is a single NP and a single NP receptor in the jawless Agnathan fish [50,51]. Both the ligand and the receptor family differentiated during fish evolution in response to selection pressure to achieve body fluid homeostasis in osmotically variable aquatic environments. The medaka fish and puffer fish genomes contain six NP genes [51] together with two GCA and one GCB receptor genes [52]. This level of complexity in natriuretic peptide signalling has not been retained in mammals, as mammalian genomes possess three NP genes and two rGC natriuretic receptor genes. It has been proposed that a reduction in the number of natriuretic ligands and their receptors in amniotes was associated with the transition from an osmotically variable aquatic environment to dry land, where the most important aspect of body fluid regulation became the retention of water [51]. These vertebrate natriuretic rGCs are found within a larger clade, which also contains insect rGC sequences (Figure 2). Based on the available data it is impossible to determine if these insect receptors are involved in insect natriuresis. However, the *Bombyx mori *rGC found in this clade is expressed in the antennal lobe [11], therefore its function is more likely to be involved in chemoreception. The GC-C receptor, although also involved in salt regulation in the intestine, seem to have an independent phylogenetic origin from the natriuretic GC receptors.

In mammals the functional sGC unit is an α/β heterodimer which binds one heme group per dimer. Selective binding of NO at the heme iron activates the enzyme to convert GTP to the second messenger cGMP. The selectivity displayed by NO sensitive sGC is remarkable, considering that the heme in sGC is identical to that in the O_2 _storage and transport proteins and that the concentration of O_2 _is higher by 3 orders of magnitude than NO in eukaryotic cells [30]. Our phylogenetic reconstruction shows the existence of a basal group of NO insensitive insect and nematode sGCs which are activated by O_2_, implying that the primordial eukaryotes probably utilized sGC as an O_2 _sensor. The *M. sexta *MsGC-3 subunit from the basal clade forms active sGC homodimers [26,44], as also does its *D. melanogaster *homologue Gyc-88E [43,53], while the *C. elegans *genome lacks sGC α-subunit genes. However, there is genetic evidence that the β-subunit like sGC proteins GCY-35 and GCY-36 of *C. elegans *can function as α/β-like heterodimers [54]. Thus the initial form of the eukaryote O_2 _sensitive sGC may have been a homodimer, with subsequent evolution and diversification of the α and β lineages leading to the formation of heterodimers, a shift to NO sensitivity and diversification of function.

Phylogenetic analyses suggest that that the sGC H-NOB domain was acquired by horizontal transfer from a cyanobacterial source [22]. The H-NOB domain of facultative aerobic bacteria and the cyanobacteria is predicted to bind NO [30] and it is clear from Figure 7 that both *Anabaena *and *Nostoc *lack the Tyr-140 residue which is essential for the stabilisation of O_2 _binding in the *T. tengcongensis *H-NOB. Thus the primordial eukaryotes probably obtained a cyanobacterial H-NOB domain which was sensitive to NO; substitution of one of the hydrophobic residues in the distal heme-binding pocket by a Tyr residue in the primordial animal sGC H-NOB domain would have changed the ligand specificity to O_2_. Subsequent replacement of the Tyr residue in the distal heme pocket by an I1e residue in the β-1 and β-2 sGC lineages would have regenerated an NO sensitive sGC. Once NO sensitive sGCs evolved, they acquired additional physiological functions, including regulation of vascular and non vascular smooth muscle relaxation and vascular homeostasis [55], antimicrobial and anti tumor activity [56] as well as roles in neuronal survival and synaptic maintenance [57].

Lineage specific expansion of both the rGC and sGC gene families has occurred in nematodes, the largest of these is a rGC expansion comprising 21 genes (Figure 2). All seven *C. elegans *sGC are expressed in sensory neurons [15,29] but in addition *gcy-35 *has a wider distribution, being also expressed in pharyngeal and body wall muscles and the excretory cell [29]. The available expression patters for the rGC genes also implicate them in nervous system function. Previously it has been demonstrated that five rGCs are specifically expressed in sensory neurons or interneurons in *C. elegans *[15]; expression of the rGCs *gcy-5*, *gcy-6 *and *gcy-7 *was observed in the ASE neurons which detect water soluble cues. The rGC gene, *odr-1*, is expressed in a subset of chemosensory neurons and is essential for responses to all volatile odorants sensed by the AWC neurons [16]. Similarly the rGC gene *daf-11 *is expressed in a number of sensory neurons and *daf-11 *mutants have defects in dauer pheromone response and in their ability to detect certain odors. The sGC, GCY-35, has been shown to mediate oxygen sensation in *C. elegans *[29]. Thus both sGCs and rGCs have been shown to have central roles in chemosensation in *C. elegans*. The chemosensory system of nematodes displays many differences from the olfactory systems of vertebrates and insects [58], largely resulting from the relatively small number of olfactory neurons in nematodes. *C. elegans *has only twelve pairs of sensory neurons within each of its two olfactory amphid organs. However the relative lack of anatomical complexity in the nematode sensory nervous system appears to have been compensated during nematode evolution by an increased functional complexity and multitasking capacity of individual sensory neurons [58]. For example, individual olfactory neurons express multiple odor receptors, multiple heterotrimeric G protein α subunits, multiple GCs and they display a wide range of other, often-novel, mechanisms for signal integration within individual neurons. In consequence, lineage specific gene expansions are particularly noticeable in nematodes for neuronal gene families. For example the largest and most diverse nicotinic acetylcholine receptor gene family is that of *C. elegans *[59]; novel families of potassium channels have been identified in *C. elegans *[60]; a nematode specific expansion in the heterotrimeric G protein α-subunit gene family has been documented [61] and G protein coupled chemoreceptor genes comprise the largest gene family in *C. elegans *[62]. Additionally, asymmetric expression patterns of neuronal genes increases the discriminatory power and olfactory potential of *C. elegans *[63]. One such example is the asymmetric expression of rGC genes in the bilaterally symmetrical ASE taste receptor neurons [64]. In adult worms the rGC genes *gcy-6 *and *gcy-7 *are only expressed in left sided ASE neurons, whereas *gcy-5 *is expressed only in right hand sided ASE neurons. This asymmetry of rGC expression correlates with a functional asymmetry of the left and right ASE neurons and thereby increases the odor discrimination capacity of the nematodes [65].

The chromosomal position of all "paranome genes" in *C. elegans *has recently been reported [66]. The "paranome" is defined as the set of all duplicate genes in a genome [66]. These authors found that duplications within the *C. elegans *genome are generally intrachromosomal while in *S. cerevisiae *they are usually interchromosomal. Chromosome V appears to have undergone a high degree of self-duplication in *C. elegans*, as 48.9% of its 4,792 genes are paranome members. *C. elegans *chromosomes II and IV also have a high percentage of paranome genes: 31.6% and 33% respectively [66]. Three of the seven sGCs and seven of the 25 receptor GCs reside on chromosome V, a finding that supports these previous observations [66]. The chromosomal location of all GCs in *C. elegans *is closely correlated with phylogenetic position on our reconstructed trees (this is especially true for rGCs).

Analysis of eukaryotic proteomes has shown that all but a small proportion of the eukaryotic protein repertoire is formed from protein domains which have been extant since the origin of eukaryotes [67]. This trend is also apparent for the sGC and rGC proteins where the prokaryote progenitors of the Class III purine nucleotide cyclase, the H-NOX family of domains and the kinase homology domains have been identified. The conservation of the linear order of the individual domains of the rGC and sGC proteins, respectively, together with the extent of sequence identity across the entire lengths of these proteins from both vertebrate and invertebrate animals strongly suggests that each protein family is of monophyletic origin. The rGC and sGC proteins detected in the protistan systems investigated to date are more closely related in terms of sequence identity and domain topology to adenylyl cyclases (ACs) [38,68,69]. Metazoan membrane bound ACs are composed of two membrane domains, each consisting of six transmembrane helices. Each membrane domain is followed by a catalytic domain and the two catyalytic domains function as a functional heterodimer with a single catalytic pocket [70]. In *Dictyostelium discoideum *the GC gene, DdGCA, encodes a protein with 12 transmembrane helices and two cyclase domains, [67], a configuration also found in the GCs of malaira parasite *Plasmodium falciparum *and the ciliates *Paramecium *and *Tetrahymena *[38]. By contrast, membrane bound GCs of metazoans have only one single helix transmembrane domain and one cyclase domain. Thus the animal rGC and sGC families appear to have evolved after the divergence of the animal and protistan lineages. No rGC and sGC sequence information is currently available for the parazoa, so it is not known if the animal GCs evolved before the divergence of the parazoa and eumetazoa. The evolution of novel genes by the amalgamation of individual functional domains has been a frequent route for the emergence of new signal transduction and cell communication mechanisms in metazoans [71-75]. The modular arrangement of the sGC and rGC proteins has facilitated the evolution of novel signalling pathways in animal lineages through modifications to the receptor domains and by combining the cGMP product of GC activation with distinct downstream effectors such as cGMP dependent protein kinases, cGMP-gated ion channels and phosphodiesterases. The sGC, while sensitive only to membrane permeant NO or O_2_, has coevolved with a very sensitive and complex NO production system which provides a very effective local cell-to-cell signalling system. Our phylogenetic analysis reveals that once the rGC and sGC multidomain proteins had evolved in the animal lineage subsequent gene duplications, tissue specific expression patterns and lineage specific expansions resulted in the evolution of new networks of interaction and new biological functions associated with the maintenance of organismal complexity and homeostasis.

## Conclusion

GCs are responsible for the production of the secondary messenger cGMP, which plays important roles in a variety of physiological responses such as vision, olfaction, muscle contraction, homeostatic regulation, cardiovascular and nervous function. There are two types of GCs in animals, soluble sGCs which are found ubiquitously in cell cytoplasm, and receptor GC forms which span cell membranes. We have reconstructed molecular phylogenies for both sGC and rGC proteins. The most notable features of the resulting phylogenies are the number of lineage specific rGC and sGC expansions that have occurred during metazoan evolution. Among these expansions is a large nematode specific rGC clade; a vertebrate specific expansion in the natriuretic receptors GC-A and GC-B; a vertebrate specific expansion in the guanylyl GC-C receptor, an echinoderm specific expansion in the sperm rGC genes and a nematode specific sGC clade. The nematode specific GC genes identified within this study have expression and localisation patterns specific to sensory neurons. This expansion of the molecular diversity in individual neurons may compensate for the relative lack of anatomical complexity in the nematode sensory nervous system.

Our phylogenetic reconstruction also shows the existence of a basal group of nitric oxide (NO) insensitive insect and nematode sGCs which are activated by O_2_. This suggests that the primordial eukaryotes probably utilized sGC as an O_2 _sensor, and that the ligand specificity of sGC later switched to NO which provides a very effective local cell-to-cell signalling system.

Our phylogenetic analysis of animal and bacterial H-NOB domain sequences supports the hypothesis [22] that this domain originated from a cyanobacterial source. It has been shown that the introduction of a polar Tyr residue into the non polar distal pocket of the H-NOB domain is sufficient to change its binding specificity from NO to O_2_. Our alignment of H-NOB domain sequences shows that non-polar residues only line the predicted distal pocket region of the β-1 and β-2 sGC sequences, which bind NO. However, all sequences from the basal sGC clade and the nematode specific clade which bind O_2 _have a Tyr residue in the predicted distal pocket region (with the exception of GCY-37 which has a conservative Phe substitution). These observations support the hypothesis that the presence of a Tyr residue in the distal pocket of the H-NOB domain is necessary for O_2 _binding, and is used to kinetically distinguish between NO and O_2 _[31,76].

## Methods

### Sequences and alignments

sGC and rGC homologues were located by performing multiple BLASTP [77] searches with a cut off expectation value (E-value) of 10^-7 ^against GenBank. In each case putative *C. elegans *GC proteins were used as the query sequence. The complete pufferfish and honeybee genomes are not yet deposited in GenBank. These were obtained from ensembl [78]. Multiple BLASTP searches were performed again with putative *C. elegans *GC proteins used as query sequences against these genomes, sequences with significant E values were added to our dataset for phylogenetic analysis. In total, 38 sGCs and 82 rGCs from many diverse genera were located (see additional file 1 for accession numbers). Both sets of proteins were aligned using ClustalW 1.81 [79] using the default settings. All alignments were corrected for obvious alignment ambiguity. The resultant sGC alignment contained 1714 aligned positions and the rGC alignment contained 2021 aligned positions.

A previous study has shown that echinoderms have many diverse GC isoforms [25]. To investigate the relationships between these echinoderm sequences and our dataset, we aligned the corresponding region of the rGC cyclase domain of all human, mouse, *D. melangaster *and *A. gambiae *GC genes with the echinoderm sequences. We also aligned the cyclase domain of particular nematode guanylyl cyclase proteins (GCY-11, GCY-12, GCY-15, GCY-21) to the echinoderm cyclase domain. The resultant alignment was edited by eye.

The H-NOB domain of various aerobic and anaerobic bacteria were compared to eukaryotic H-NOB domains. Domains were aligned using ClustalW 1.81 and edited by eye (Figure 7). Accession numbers for additional bacterial sequences can be found in additional file 1.

### Gene tree reconstruction

Bayesian trees for the sGC and rGC proteins were constructed using MRBAYES 3.0B4 [80]. Among site rate variation was modelled by a discrete approximation to a gamma distribution (4 categories) and a proportion of invariant sites, the shape parameter and proportion of invariant sites was allowed to vary through the Markov Chain Monte Carlo (MCMC) chain. In total, four MCMC chains were run for 3 million generations, trees were sampled every 100^th ^generation. Plots of likelihood versus generation for both gene families revealed that all chains reached stationarity after 200,000 generations therefore 200,000 trees were discarded as a *burnin *for both alignments. Clade probabilities for each phylogeny were determined using the *sumt *command of MRBAYES 3.0B4.

For completeness we also constructed maximum likelihood phylogenies for both protein families. Appropriate protein models were selected for each family using the software program MODELGENERATOR [81]. One hundred bootstrap replicates were then carried out with the appropriate protein model using the software program PHYML [82] and summarised using the majority-rule consensus method. Supports from the maximum likelihood analyses were comparable to the Bayesian analyses. Phylogenetic trees for both the partial cyclase domain (Figure 4) and H-NOB domain (Figure 6) were constructed in an identical fashion.

### EST database searches

Using each *C. elegans *GC gene as a query sequence, we performed exhaustive TBLASTN [77] database searches with a cut off expectation value of 10^-7 ^against the nematode EST database NEMBASE [83] and the *Caenorhabditis briggsae *genome at Wormbase [84]. The version of NEMBASE used contained 130,184 clustered ESTs from 37 different nematode species from four of the five major nematode clades [85]. All statistically significant EST sequence hits were extracted and subsequently searched locally against the *C. elegans *proteome [86] using BLASTX with a cut off expectation of 10^-7^. Significant hits were confirmed by manual inspection of BLAST alignments. The purpose of this approach was to confirm orthology between the nematode EST sequences and the *C. elegans *protein sequences. The presence or absence of nematode specific genes within the 37 species of nematodes found in NEMBASE was noted. The *Brugia malayi *genome was obtained from The Institute of Genomic Research [87]. Database searches of this genome using BLASTP revealed that this species contains a number of putative GC genes (Table 1).

Using the same methodology as above, nematode specific genes were used to search the schistosome [88] and tardigrade [89] EST databases. No orthologues were found for the nematode specific group of GC genes in these additional database searches.

## Authors' contributions

All authors were involved in the design phase. DMO sourced all known nematode guanylyl cyclase proteins and DF located subsequent proteins from GenBank, completed genomes and EST databases. DF and DMO performed all EST database searches. DF and DMO performed the phylogenetic analysis and chromosomal locations of nematode genes. AMB directed the project and provided advice on all analyses. All authors were involved in the drafting of the manuscript and approved the final manuscript.

## Supplementary Material

Additional File 1 Accession numbers are given for all sequences used in this analysis. All accession numbers are linked to GenBank files except those of *Fugu rubripes *and *Apis mellifera *which relate to ENSEMBL accessions. Click here for file
