# Supplementary material for: Multiple lineage specific expansions within the guanylyl cyclase gene family
Source: BMC Evol Biol. 2006 Mar 20;6:26. doi: 10.1186/1471-2148-6-26 (PMC1435932; doi:10.1186/1471-2148-6-26)
Supplement: Additional File 1 — Accession numbers are given for all sequences used in this analysis. All accession numbers are linked to GenBank files except those of Fugu rubripes and Apis mellifera which relate to ENSEMBL accessions. [file 1471-2148-6-26-S1.pdf]

Accession Numbers

The accession numbers for the sequence data used in this analysis from Genbank release 147.0 and genomes retrieved from Ensembl are as follows: Organisms whose genomes have been completely sequenced are denoted with an asterisk.

Insoluble:

Homo sapiens *
GCA [Genbank:NP_000897]
GCB [Genbank:NP_003986]
GCC [Genbank:NP_004954]
GCD [Genbank:NP_000171]
GCF [Genbank:NP_001513]

Mus musculus *
GCA [Genbank:NP_032753]
GCB [Genbank:NP_776149]
GCC [Genbank:XP_132928]
GCE [Genbank:NP_032218]
GCF [Genbank:NP_001007577]
GCG [Genbank:XP_129326]

Gallus gallus *
a [Genbank:XP_417281]
b [Genbank:XP_416207]
c [Genbank:XP_429192]

Caenorhabditis elegans *
gcy-1 [Genbank:NM_063638]
gcy-2 [Genbank:NM_063637]
gcy-3 [Genbank:NM_063636]
gcy-4 [Genbank:NM_063817]
gcy-5 [Genbank:NM_063818]
gcy-6 [Genbank:NM_073249]
gcy-7 [Genbank:NM_001028784]
gcy-8 [Genbank:NM_068923]
gcy-9 [Genbank:NM_077496]
gcy-11 [Genbank:NM_078354]
gcy-12 [Genbank:NM_062594]
gcy-13 [Genbank:NM_073696]
gcy-14 [Genbank:NM_074259]
gcy-15 [Genbank:NM_062067]
gcy-17 [Genbank:NM_058542]
gcy-18 [Genbank:NM_070048]
gcy-19 [Genbank:NM_062090]
gcy-20 [Genbank:NM_074700]
gcy-21 [Genbank:NM_061923]
gcy-22 [Genbank:NM_075617]
gcy-23 [Genbank:NM_067908]
gcy-25 [Genbank:NM_070490]
gcy-27 [Genbank:NM_070709]
odr-1 [Genbank:NM_077865]

***Drosophila melanogaster* ***
a [Genbank:NP_611532]
b [Genbank:NP_650505]
c [Genbank:NP_649477]
d [Genbank:AAR31133]
e [Genbank:CAA51319]
f [Genbank:NP_648653]

***Anopheles gambiae* ***
a [Genbank:EAA14802]
b [Genbank:EAA00176]
c [Genbank:EAA00177]
d [GenbankEAA03567]
e [Genbank:EAA10186]
f [Genbank:EAA03699]

Fugu rubripes *
a [ENSEMBL:SINFRUG00000121899]
b [ENSEMBL:SINFRUG00000131285]
c [ENSEMBL:SINFRUG00000133934]
d [ENSEMBL:SINFRUG00000140601]
e [ENSEMBL:SINFRUG00000145293]
f [ENSEMBL:SINFRUG00000151673]
g [ENSEMBL:SINFRUG00000155431]
h [ENSEMBL:SINFRUG00000158909]

Oryzia latipes
GC1 [Genbank:BAA20563]
GC2 [Genbank:BAB21106]
GC3 [Genbank:BAA19205]
GC4 [Genbank:BAA19206]
GC5 [Genbank:BAA19207]
GC6 [Genbank:BAA76279]
GC7 [Genbank:BAB21105]
GC8 [Genbank:BAB21378]
GC9 [Genbank:BAD11374]
OlGCR2 [Genbank:BAA76301]

***Apis mellifera* ***
a [ENSEMBL:ENSAPMG00000005958]
b [ENSEMBL:ENSAPMG00000006282]
c [ENSEMBL:ENSAPMG00000006724]
d [ENSEMBL:ENSAPMG00000006725]

Echinoderms
Asterias amurensis [Genbank:BAB85468]
Brissus agassizii [Genbank:BAA75197]
Diadema setosum [Genbank:BAA85332]
Arbacia punctulata [Genbank:OYURGA]
Hemicentrotus pulcherrimus [Genbank:BAA04660]
Strongylocentrotus purpuratus [Genbank:NP999705]
Stichopus japonicus [Genbank:BAA75224]

Miscellaneous
Bactrocera dorsalis [Genbank:AAM94353]
Manduca sexta [Genbank:AAN16469]

Bombyx mori [Genbank:BAB32672]

Soluble:

Homo sapiens *
beta-1 [Genbank:NP_000848]
beta-2 [Genbank:NP_004120]
alpha-1 [Genbank:NP_000847]
alpha-2 [Genbank:NP_000846]

Mus musculus *
beta-1 [Genbank:NP_059497]
beta-2 [Genbank:NP_766398]
alpha-1 [Genbank:NP_068696]
alpha-2 [Genbank:XP_150145]

Caenorhabditis elegans *
gcy-31 [Genbank:NM_001029719]
gcy-32 [Genbank:NM_074051]
gcy-33 [Genbank:NM_073609]
gcy-34 [Genbank:NM_073918]
gcy-35 [Genbank:NM_060943]
gcy-36 [Genbank:NM_078156]
gcy-37 [Genbank:NM_067770]

Gallus gallus*
alpha-1 [Genbank:XP_420375]
alpha-2 [Genbank:XP_420375]
beta-2 [Genbank:XP_426684]

Apis mellifera *
alpha-1 [Genbank:BAD6682]
beta-1 [Genbank:AAV34676]

Fugu rubripes *
beta-1 [Genbank:BAB60908]
alpha-2 [Genbank:BAC76397]
alpha-1 [Genbank:BAB60907]
beta-2 [ENSEMBL:SINFRUG00000128383]

Drosophila melanogaster *
Gyc88E [Genbank:AAF55135]
Gyc-89Da [Genbank:AAF55322]
Gyc-89Db [Genbank:AAF55323]
beta [Genbank:AAF57119]
alpha [Genbank:AAF56917]

Limax marginatus
beta-1 [Genbank:BAC80152]
beta-2 [Genbank:BAC80153]
alpha [Genbank:BAC80151]

Manduca sexta
beta-1 [Genbank:AAC61264]
beta-2 [Genbank:AAD09836]
alpha-1 [Genbank:AAC61263]

Miscellaneous
Bactrocera dorsalis alpha-1 [Genbank:AAP85539]
Aplysia californica [Genbank:AAK97794]
Anopheles gambiae beta [Genbank:AAC47144]

Additional bacterial sequences for Figure 7

Shewanella oneidensis [Genbank:ZP_00111432],
Thermoanaerobacter tengcongensis [Genbank:NP_622340],
Magnetococcus [Genbank:EAN29238],
Caulobacter crescentus [Genbank:AAK24954],
Legionella pneumophila [Genbank:AAU28519],
Rhodobacter sphaeroides [Genbank:ABA79585]
Clostridium acetobutylicum [Genbank:NP_349837],
Nostoc [Genbank:ZP_00111432],
Anabaena [Genbank:BAB73977]
